# Gene Expression Profile of the Hypothalamus in DNP-KLH Immunized Mice Following Electroacupuncture Stimulation

**DOI:** 10.1093/ecam/nep222

**Published:** 2011-06-05

**Authors:** Sun Kwang Kim, Jeungshin Kim, Eunjung Ko, Hyunseong Kim, Deok-Sang Hwang, Sanghoon Lee, Yonghyeon Baek, Byung-Il Min, Sangsoo Nam, Hyunsu Bae

**Affiliations:** ^1^Department of Physiology, College of Oriental Medicine, Kyung Hee University, #1 Hoegi-dong, Dongdaemoon-gu, Seoul 130-701, Republic of Korea; ^2^BK21 Oriental Medical Science Center, Republic of Korea; ^3^Acupuncture & Meridian Science Research Center, Republic of Korea; ^4^Department of Acupuncture & Moxibustion, College of Oriental Medicine, Kyung Hee University, #1 Hoeki-dong, Dongdaemoon-gu, Seoul, 130-701, Republic of Korea; ^5^Department of Oriental Gynecology, College of Oriental Medicine, Republic of Korea; ^6^Department of Physiology, College of Medicine, Kyung Hee University, Seoul, Republic of Korea

## Abstract

Clinical evidence indicates that electroacupuncture (EA) is effective for allergic disorder. Recent animal studies have shown that EA treatment reduces levels of IgE and Th2 cytokines in BALB/c mice immunized with 2,4-dinitrophenylated keyhole limpet protein (DNP-KLH). The hypothalamus, a brain center of the neural-immune system, is known to be activated by EA stimulation. This study was performed to identify and characterize the differentially expressed genes in the hypothalamus of DNP-KLH immunized mice that were stimulated with EA or only restrained. To this aim, we conducted a microarray analysis to evaluate the global gene expression profiles, using the hypothalamic RNA samples taken from three groups of mice: (i) normal control group (no treatments); (ii) IMH group (DNP-KLH immunization + restraint); and (iii) IMEA group (immunization + EA stimulation). The microarray analysis revealed that total 39 genes were altered in their expression levels by EA treatment. Ten genes, including T-cell receptor alpha variable region family 13 subfamily 1 (Tcra-V13.1), heat shock protein 1B (Hspa1b) and 2′–5′ oligoadenylate synthetase 1F (Oas1f), were up-regulated in the IMEA group when compared with the IMH group. In contrast, 29 genes, including decay accelerating factor 2 (Daf2), NAD(P)H dehydrogenase, quinone 1 (Nqo1) and programmed cell death 1 ligand 2 (Pdcd1lg2) were down-regulated in the IMEA group as compared with the IMH group. These results suggest that EA treatment can modulate immune response in DNP-KLH immunized mice by regulating expression levels of genes that are associated with innate immune, cellular defense and/or other kinds of immune system in the hypothalamus.

## 1. Introduction

Several clinical studies have previously suggested that electroacupuncture (EA) is effective for the treatment of various types of allergic disorders, including asthma and chronic urticaria [1–4]. It is generally accepted that hyperproduction of IgE in response to stimulation by Th2 cytokines (i.e., IL-4, IL-5 and IL-13) promotes the development of these allergic disorders [5, 6]. Our previous study demonstrated that successive EA at the ST36 acupoint reduces serum levels of IgE in BALB/c mice that have been immunized with 2,4-dinitrophenylated keyhole limpet protein (DNP-KLH) via suppression of the production of Th2 cytokines [7]. Furthermore, it has been shown that these effects of EA are mediated, at least in part, by *α*-adrenergic receptors [8] and are acupoint (ST36) specific [9].

The hypothalamus is the primary center for neuroendocrine-immune modulation [10]. The paraventricular nucleus of the hypothalamus (PVN) represents an integral part of the neuroendocrine circuit that modulates immune function. Moreover, stimulation of the ventral nucleus of the hypothalamus (VMH) stimulation enhances the immune function associated with the lateral hypothalamus (LH) and ventral tegmental area (VTA) of the brain. Interestingly, previous brain imaging studies of animals and humans have shown that EA treatment affects the neuronal activity of the hypothalamus [11–16]. Although it has also been reported that EA influences the immunomodulatory effects of the hypothalamus [17, 18], the studies that have been conducted to date have only investigated the effects of EA on normal rats.

Oligonucleotide microarray analysis is the most powerful tool for functional genomics that provides direct information about mRNA expression levels from a large number of genes. This method has been used to find the genes correlated with the pathogenesis of diseases and to assess the drug actions on the diseases [19, 20]. To examine if and how EA treatment modulates immune function of the hypothalamus at transcriptional level in a Th2-skewed condition, we conducted a microarray analysis on the hypothalamus of DNP-KLH immunized mice that were stimulated with EA.

## 2. Methods

### 2.1. Animals

Eight-week-old female BALB/c mice were purchased from Orient Bio (Sungnam, Korea) and housed in an air-controlled, pathogen-free animal facility under a 12-h light/dark cycle at 23 ± 2°C. Mice were provided with a standard laboratory diet and water *ad libitum*. All procedures involving animals were conducted in accordance with the NIH guidelines.

### 2.2. Experimental Groups

Mice were randomly divided into the following 3 groups (*n* = 6–10/group): (i) NC group (normal control, no treatments for 2 weeks, except for i.p. injection of saline on the 1st and 8th experimental day); (ii) IMH group (immunization with 2,4-dinitrophenylated keyhole limpet protein (DNP-KLH) on the 1st and 8th experimental day and being held in a holder restraint for 20 min a day for 2 weeks; (iii) IMEA group (immunization on the 1st and 8th experimental day + daily EA stimulation at the ST36 acupoint while being held in a holder restraint for 20 min a day for 2 weeks).

### 2.3. EA Stimulation and DNP-KLH Immunization

EA stimulation was performed as previously described [7]. Briefly, a pair of stainless steel needles (0.2 mm in diameter and 3 cm long) was inserted into the ST36, which is located at the anterior tibial muscle, 5 mm laterally and lower from the anterior tubercle of the tibia, and at a point 5 mm distal from the first needle. The anode and cathode leads from an electrical stimulator were then connected to the two acupuncture needles, after which train-pulses (1 Hz, 0.25 ms pulse width, 3–5 V) were applied for 20 min. The stimulation intensity was determined to be the minimum voltage that induced a moderate muscle contraction.

All mice, except for the NC group, were immunized intraperitoneally with 4 *μ*g of DNP-KLH (Calbiochem, Gibbstown, NJ, USA) and 4 mg of aluminum hydroxide (Sigma, St Louis, MO, USA) on the 1st and 8th day during the 2-week experimental period. Immunization of BALB/c mice with DNP-KLH caused a significant increase in IgE and IL-4 production on the 14th day after first immunization; therefore, EA stimulation was conducted daily for 2 weeks [7].

### 2.4. Measurements of IgE in Serum Using ELISA

Serum was obtained from all groups of mice on the final experimental day and then stored at −20°C until analysis. The Serum level of IgE was measured using a quantitative sandwich enzyme-linked immunoassay kit (BD, Franklin Lakes, NJ, USA). Briefly, a 96-well microtiter plate (Coster, Cambridge, MA, USA) was incubated overnight at 4°C with anti-mouse IgE monoclonal antibody in coating buffer. The plate was then washed with PBS containing 0.05% tween 20 (Sigma, USA) and blocked with 5% FBS in PBS for 1 h at room temperature. Subsequently, 100 *μ*l of sample were loaded onto the plate, which was then incubated for 2 h at room temperature. Next, secondary peroxidase labeled biotinylated anti-mouse IgE monoclonal antibody in 5% FBS in PBS diluent was added and the plate was incubated for an additional 1 h. The plates were then treated with TMB substrate solution for 30 min, after which the reaction was stopped by the addition 50 *μ*l of 2N H_2_SO_4_ stop solution per well. Finally, the optical density at 450 nm was measured in a microplate reader (TECAN, Durham, NC, USA).

### 2.5. Oligonucleotide Chip Microarray

The hypothalamuses were obtained from all groups of mice on the final experimental day and then stored at −80°C until analysis. Total RNA was extracted from the hypothalamus by using TRIZOL reagent, and DNase I (Invitrogen Life Technologies, Rockville, MD, USA) was treated to eliminate genomic DNA contamination. Oligonucleotide chip microarray was performed using single round RNA amplification protocols, following the Affymetrix specifications (Affymetrix GeneChip Expression Analysis Technical Manual). Briefly, 3 *μ*g of total RNA was used to synthesize first-strand complementary DNA (cDNA) using oligonucleotide probes with 24 oligo-dT plus T7 promoter as primers (Proligo LLC, Boulder, CO, USA) and the Superscript Choice System (Life Technologies, Invitrogen, Milan, Italy). Following double-stranded cDNA synthesis, the products were purified by phenol-chloroform extraction, after which biotinylated antisense complimentary RNA (cRNA) was generated through *in vitro* transcription using a BioArray RNA High-Yield Transcript Labeling Kit (ENZO Life Sciences Inc., Farmingdale, NY, USA). Next, the biotinylated labeled cRNA was fragmented, and then 10 g of the total fragmented cRNA was hybridized to the GeneChip Mouse Gene 1.0 ST Array (P/N901178, Affymetrix Inc., Santa Clara, CA, USA). The Affymetrix Fluidics Station 400 was then used to wash and stain the chips, after which the nonhybridized target was removed. The samples were then incubated with a streptavidin-phycoerythrin conjugate to stain the biotinylated cRNA. Next, the staining was amplified using goat IgG as a blocking reagent and biotinylated antistreptavidin antibody (goat), followed by a second staining step using a streptavidin-phycoerythrin conjugate. The fluorescence was then detected using the Genechip System Confocal Scanner (Hewlett-Packard, Santa Clara, USA), and analysis of the data contained on each GeneChip was conducted using the GeneChip 3.1 software produced by Affymetrix using the default settings. Global scaling was then used to compare the chips, with all probe sets being scaled to a user-defined target intensity of 150.

### 2.6. Data Analysis

One-way ANOVA followed by Newman–Keuls multiple comparison test was used for statistical analysis of the IgE data. In all cases, *P *< .05 was considered significant. Data are presented as the mean ± SEM.

For analysis of the microarray data, the MAS5 algorithm was used to evaluate the expression signals generated by the Affymetrix Mouse Gene 1.0 ST Array. Global scaling normalization was then performed, after which the normalized data were log-transformed using base 2. Next, a fold change and a Welch *t*-test were applied to select the differentially expressed genes (DEGs) using a fold change threshold of 1.2 and a *P *< .05 to indicate significance. Each probe set used in the Affymetrix GeneChip produces a detection call, with P (present call) indicating good quality, M (marginal call) indicating intermediate quality and A (absent call) indicating relatively low reliability. Therefore, probe sets that resulted in A calls were removed to filter false positives. A volcano plot was used to better visualize and compare the two DEG methods. The 1.2-fold DEGs were clustered using the GenPlex^TM^ v2.3 software (ISTECH Inc., Ilsan, Korea) using hierarchical clustering with Pearson correlation as a similarity measure and complete linkage as the linkage method. In addition, gene ontology significance analysis was conducted to investigate the functional relationships among the 1.2-fold DEGs using high-throughput GoMiner. The 1.2-fold DEGs were then mapped to the relevant pathways using the GenPlex v2.4 software (ISTECH Inc., Ilsan, Korea). The pathway resources were provided by the KEGG database.

## 3. Results

### 3.1. IgE Levels in DNP-KLH Immunized Mice Following EA Stimulation

**Figure 1:**
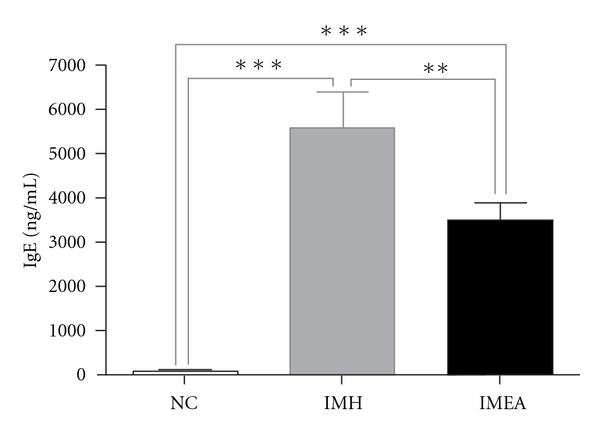
Comparison of serum IgE levels in the three experimental groups. The amounts of serum IgE were measured by ELISA. Data are presented as the mean ± SEM. **P <  .01, ***P <  .001 between the two indicated groups as determined by the Newman-Keuls multiple comparison test following one-way ANOVA. NC (*n* = 7, no treatments for 2 weeks, except for i.p. injection of saline on the 1st and 8th experimental day); IMH (*n* = 6, immunization on the 1st and 8th experimental day + daily holder restraint); IMEA (*n* = 6, immunization on the 1st and 8th experimental day + daily EA stimulation at the ST36 acupoint under holder restraint).

BALB/c mice were immunized with DNP-KLH and aluminum hydroxide once a week. In the IMEA group, 20-min EA stimulation at the ST36 acupoint under restraint with an acryl holder was performed daily for 2 weeks, whereas mice in the IMH group were placed in the holder restraint for 20 min a day for 2 weeks, but not treated. After the 2-week experimental period, the serum levels of IgE were measured.  Figure 1 shows serum levels of IgE in mice. The IgE levels in the IMH and the IMEA group were significantly increased when compared with the IgE levels of the NC group (*P *< .001). However, EA treatment significantly reduced the elevated IgE levels (*P *< .01, IMEA versus IMH).

### 3.2. DEGs in the Hypothalamus of Mice in the NC, IMH and IMEA Groups

The genes that were differentially expressed among the groups are listed in Table 1 In addition, the genes are grouped into functional categories and pathways based on the KEGG database in Table 2. There were 10 genes that were down-regulated in the IMH group (versus NC) and up-regulated in the IMEA group (versus IMH). Conversely, 17 genes were up-regulated in the IMH group (versus NC) and down-regulated in the IMEA group (versus IMH). Additionally, 12 genes were not significantly changed in the IMH group (versus NC), but were down-regulated in the IMEA group (versus IMH).

### 3.3. Clustering Analysis and Gene Ontology Analysis

**Figure 2:**
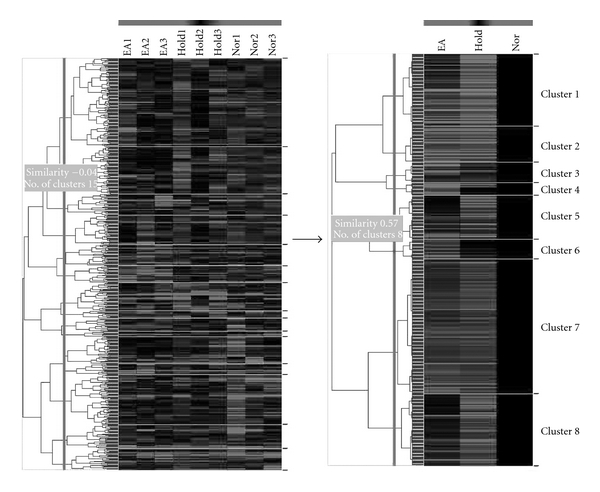
Clustergram of up- and down-regulated genes in DNP-KLH immunized BALB/c mice that were subjected to EA stimulation. The results shown are representative of three independent samples collected for each treatment. Each gene is represented by a single row of clustered boxes and each experimental sample is represented by a single column. The entire clustered image is shown on the left. The signal averages of up- and down-regulated genes are shown on the right.

**Figure 3:**
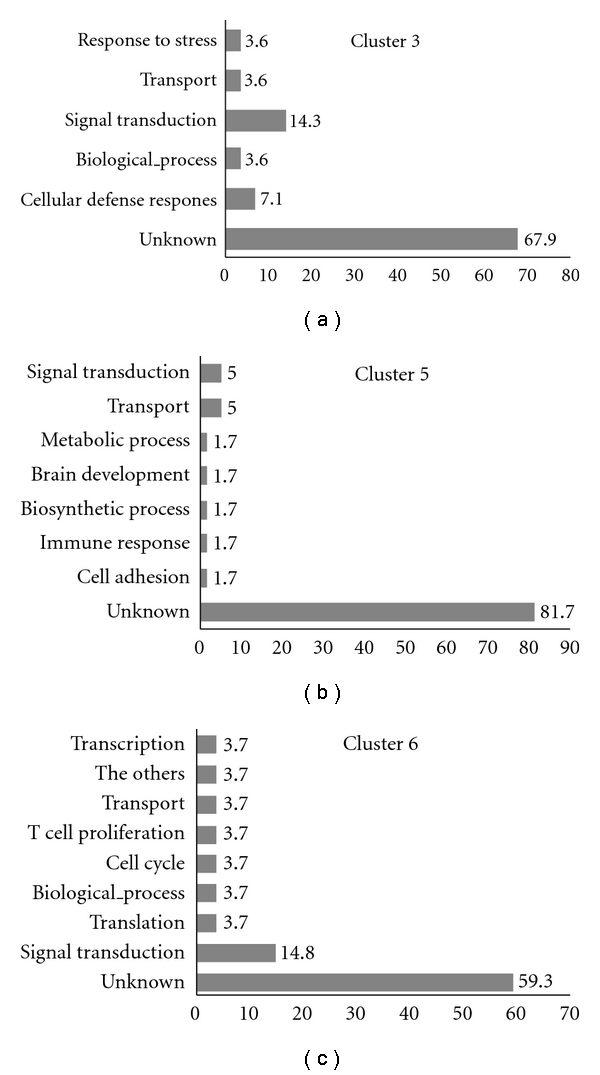
Gene ontology assignment of DEGs. Bars on Cluster 3 indicate up-regulated genes and bars on Cluster 5 and Cluster 6 indicate down-regulated genes comparing IMEA with IMH.

As shown in Figure 2, microarray data from the normal control (NC) group and the experimental (IMH and IMEA) groups were combined and clustered using hierarchical clustering. To examine the functional relationships among the 1.2-fold DEGs, the difference in gene ontology was determined using significance analysis (Figure 3). The results revealed that genes that were up- and down-regulated in the experimental groups were associated with processes including signal transduction, cellular defense response, transport, transcription, biological processes, translation, the cell cycle and T cell proliferation.

## 4. Discussion

DNP-KLH immunization is known to induce the elevation of serum IgE levels and Th2 cytokines, which play important roles in the development of various allergic disorders [7, 21]. In the present study, BALB/c mice in the IMH and IMEA groups were immunized with DNP-KLH and aluminum hydroxide. Although both groups showed a significant increase in IgE levels when compared with the NC group, IgE levels in the IMEA group were significantly lower than those in the IMH group (Figure 1). This result is consistent with the results of previous studies [7–9] and strongly suggests that EA treatment can modulate the immune response in the Th2 dominant condition.

Microarray analysis has been shown to be useful for the simultaneous profiling of global gene expression and identification of new genes or new functions of known genes [19, 20]. As described in the introduction, the hypothalamus is a primary center in the central nervous system responsible for neural-immune modulation [10] and has been shown to be affected by EA treatment [11, 12, 15]. Moreover, our recent study [18] showed that EA stimulation at ST36 can alter the transcriptional activity of the hypothalamus in normal rats. Therefore, we conducted microarray analysis of the hypothalamus of DNP-KLH immunized mice to determine if EA treatment of mice in a Th2-skewed condition can regulate gene expression in the hypothalamus.

Ten genes, including T-cell receptor alpha variable region family 13 subfamily 1 (Tcra-V13.1), trefoil factor 2 (spasmolytic protein 1) (Tff2), heat shock protein 1B (Hspa1b) and 2′–5′ oligoadenylate synthetase 1F (Oas1f), were down-regulated in the IMH group when compared with the NC group and up-regulated in the IMEA group when compared with the IMH group. Tcra-V13.1 is a subfamily of the T-cell receptor V*α* chain and contributes to the recognition of self-MHC molecules or peptides that has polymorphisms associated with diabetes [22, 23]. The expression pattern of Tff2, which is a member of the newly discovered protective factor of gastrointestinal mucous, may be associated with gastric cancer [24]. Hspa1b is involved in innate immunity and diverse biological processes including anti-apoptosis, response to stress and DNA repair [25, 26]. Oas1f is a transcription unit of a family of interferon-induced antiviral proteins that may play a role in the host's innate defense mechanism against viral infection [27, 28]. The differential expression of these genes indicates that EA treatment may increase some activities of innate immune and cellular defense at the transcriptional level.

Conversely, 17 genes including decay accelerating factor 2 (Daf2), cytochrome P450, family 2, subfamily c, polypeptide 38 (Cyp2c38), myosin light chain 2, precursor lymphocyte-specific (Mylc2pl), NAD(P)H dehydrogenase, and quinone 1 (Nqo1) were up-regulated in the IMH group when compared with the NC group and downregulated in the IMEA group when compared with the IMH group. In addition, 12 genes, including programmed cell death 1 ligand 2 (Pdcd1lg2), were downregulated in the IMEA group when compared with the IMH group, but not significantly changed in the IMH group when compared with the NC group. Daf2 is a glycosylphosphatidylinositol (GPI)-anchored membrane regulator that inhibits both the classical and alternative pathways of complement activation [29]. Cyp2c38 is a member of a superfamily of ubiquitous hemoproteins that metabolize a vast array of foreign chemicals as well as endogenous compounds [30]. Mylc2pl is a regulatory myosin light chain gene that is expressed specifically in precursor B and T lymphocytes and may play a role in lymphocyte development [31]. Nqo1 is a cytosolic enzyme that catalyzes the metabolic reduction of quinines and derivatives and is an endogenous factor in the regulation of immune response and autoimmunity [32]. Pdcd1lg2 is a ligand for programmed cell death-1, which is a receptor that plays an inhibitory role in T cell activation and has a potential role in Th2-mediated diseases [33]. As shown in Table 2 and Figure 3, it appears that no major or specific biochemical pathways are regulated by EA treatment. However, the present data suggest that EA treatment widely regulates immune response in the hypothalamus at the transcriptional level.

**Figure 4:**
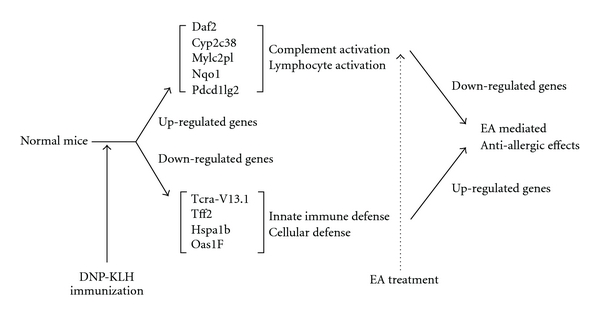
Summary of the mechanisms underlying EA-induced regulation of gene expression in the hypothalamus for anti-allergic effects.

In conclusion, EA treatment was found to reduce the serum levels of IgE that were elevated by DNP-KLH immunization in BALB/c mice. Furthermore, EA treatment altered the expression levels of 39 genes in the hypothalamus, many of which are involved in immune response (Figure 4). Taken together, these results suggest that EA treatment may induce immunomodulation by regulating gene expressions in the hypothalamus of DNP-KLH immunized mice.

## Figures and Tables

**Table 1:** Alterations in gene expression in DNP-KLH immunized BALB/c mice that were treated with EA stimulation.

Genes	Symbol	Regulation profile and average fold change	
		NC versus Hold	Hold versus EA
<Cluster 3>			
T-cell receptor alpha variable region family 13 subfamily 1	Tcra-V13.1	−0.35	0.88
Limb expression 1 homolog (chicken)	Lix1	−0.11	0.3
Trefoil factor 2 (spasmolytic protein 1)	Tff2	−0.12	0.31
Heat shock protein 1B	Hspa1b	−0.08	0.38
Olfactory receptor 1436	Olfr1436	−0.23	0.55
Olfactory receptor 1475	Olfr1475	−0.43	1.15
2′–5′ oligoadenylate synthetase 1F	Oas1f	−0.23	0.34
Olfactory receptor 689	Olfr689	−0.13	0.35
Olfactory receptor 26	Olfr26	−0.10	0.39
Probasin	Pbsn	−0.44	0.80
<Cluster 5>			
Dermatopontin	Dpt	0.15	−0.29
Decay accelerating factor 2	Daf2	0.17	−0.30
Anterior gradient homolog 3 (Xenopus laevis)	Agr3	0.13	−0.35
Secretagogin, EF-hand calcium binding protein	Scgn	0.36	−0.35
3-oxoacyl-ACP synthase, mitochondrial	Oxsm	0.22	−0.32
Empty spiracles homolog 2 (Drosophila)	Emx2	0.48	−0.45
Aldehyde dehydrogenase 3 family, member B1	Aldh3b1	0.22	−0.38
Cytochrome P450, family 2, subfamily c, polypeptide 38	Cyp2c38	0.27	−0.79
Sulfide quinone reductase-like (yeast)	Sqrdl	0.20	−0.28
Olfactory receptor 1231	Olfr1231	0.27	−0.35
Myosin light chain 2, precursor lymphocyte-specific	Mylc2pl	0.13	−0.26
NLR family, pyrin domain containing 4E	Nlrp4e	2.32	−3.96
Olfactory receptor 617	Olfr617	0.21	−0.37
Olfactory receptor 544	Olfr544	0.29	−0.37
Carboxylesterase 2	Ces2	0.09	−0.28
NAD(P)H dehydrogenase, quinone 1	Nqo1	0.17	−0.28
Sodium channel, voltage-gated, type X, alpha	Scn10a	0.17	−0.45
<Cluster 6>			
Olfactory receptor 1393	Olfr1393	−0.1	−0.47
Insulin-like growth factor 2 mRNA binding protein 1	Igf2bp1	0.05	−0.29
Cathepsin 3	Cts3	0.06	−0.39
Olfactory receptor 720	Olfr720	−0.19	−0.27
Cell division cycle 25 homolog C (S. pombe)	Cdc25c	0.05	−0.27
Programmed cell death 1 ligand 2	Pdcd1lg2	0.05	−0.34
Olfactory receptor 1310	Olfr1310	0.03	−0.55
Group specific component	Gc	−0.02	−0.32
G protein-coupled receptor, family C, group 5, member D	Gprc5d	−0.07	−0.37
Serum amyloid A 4	Saa4	−0.51	−0.53
Vomeronasal 2, receptor 65	Vmn2r65	0.05	−0.53
Intestine specific homeobox	Isx	0.05	−0.29

**Table 2:** Categorized pathways based on comparison of gene expression between IMH and IMEA groups.

KEGG Pathway	Gene counts	*P*-value
Metabolism of xenobiotics by cytochrome P450	2	5.06*E* − 05
Fatty acid biosynthesis	1	1.18*E* − 03
Biosynthesis of steroids	1	3.87*E* − 03
Phenylalanine metabolism	1	5.22*E* − 03
Histidine metabolism	1	5.89*E* − 03
Cell cycle	1	5.95*E* − 03
Neuroactive ligand- receptor interaction	1	7.11*E* − 03
Linoleic acid metabolism	1	7.23*E* − 03
Cell adhesion molecules (CAMs)	1	7.69*E* − 03
Tyrosine metabolism	1	8.74*E* − 03
Arachidonic acid metabolism	1	1.13*E* − 02
Complement and coagulation cascades	1	1.18*E* − 02
Hematopoietic cell lineage	1	1.34*E* − 02
Glycolysis/Gluconeogenesis	1	1.38*E* − 02
Leukocyte transendothelial migration	1	1.79*E* − 02
Tight junction	1	2.17*E* − 02
Focal adhesion	1	3.25*E* − 02
Regulation of actin cytoskeleton	1	3.36*E* − 02
